# Depletion of HSP60 in Microglia Leads to Synaptic Dysfunction and Depression‐Like Behaviors Through Enhanced Synaptic Pruning in Male Mice

**DOI:** 10.1111/cns.70394

**Published:** 2025-04-16

**Authors:** Wenhui Zhu, Jinlong Chang, Liusuyan Tian, Xiuyan Yang, Weifen Li

**Affiliations:** ^1^ Department of Laboratory Medicine, Guangdong Provincial Key Laboratory of Precision Medical Diagnostics, Guangdong Engineering and Technology Research Center for Rapid Diagnostic Biosensors, Guangdong Provincial Key Laboratory of Single‐Cell and Extracellular Vesicles, Nanfang Hospital Southern Medical University Guangzhou China; ^2^ Department of Physiology and Pathophysiology, School of Basic Medical Sciences Dali University Dali China; ^3^ The Seventh Affiliated Hospital Sun Yat‐Sen University Shenzhen China; ^4^ School of Pharmacy, Shenzhen University Medical School Shenzhen University Shenzhen China; ^5^ State Key Laboratory of Oncogenomics, School of Chemical Biology and Biotechnology Peking University Shenzhen Graduate School Shenzhen China

**Keywords:** depression, HSP60, microglia, phagocytosis, synapse

## Abstract

**Aims:**

Microglia, as resident macrophages in the brain, play an important role in depression. Heat shock protein 60 (HSP60), as a chaperone protein, plays a role in cell stress. However, the role of microglial HSP60 in depression remains unclear.

**Methods:**

CX3CR1‐CreER was used to generate microglial‐specific HSP60 knockout (HSP60 cKO) mice. Behavioral tests, western blotting, Golgi staining, biochemical assays, and proteomics were employed to assess depression‐like symptoms, microglial activation, and synaptic changes.

**Results:**

HSP60 cKO male mice exhibited depressive‐like behaviors, without anxiety‐like behavior, including increased immobility in the forced swimming and tail suspension tests, reduced sucrose preference, and elevated corticosterone (CORT) levels, indicating HPA axis activation. Microglial activation was confirmed by the increased expression levels of CD68 and CD86, the elevated transcription of the *cybb* gene, and reduced branch complexity. Enhanced phagocytosis of excitatory synapses, reduced dendritic spine density, and decreased glutamate levels were observed, with downregulation of synaptic proteins (AMPAR2, Synapsin‐1, PSD95), indicating dysregulated synaptic pruning. Moreover, GO analysis showed 20 significant differentially expressed proteins (DEPs) from proteomics are associated with the presynaptic endosome, which plays a crucial role in maintaining synaptic function. Treatment with PLX3397, a CSF1R inhibitor, alleviated depressive‐like behaviors and restored synaptic density in HSP60 cKO male mice.

**Conclusions:**

HSP60 deletion in microglia leads to overactivation of microglia, impaired synaptic function, and depression‐like behaviors, highlighting the importance of microglial homeostasis in mood regulation and the potential therapeutic role of microglial modulation.

## Graphical abstract

Microglial HSP60 deficiency could activate microglia, which promoted excessive pruning of excitatory neurons and decreased the level of glutamate, leading to synaptic dysfunction, resulting in depression‐like behavior of the male mice.

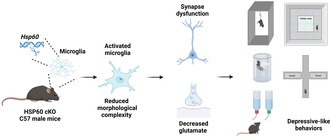

## 1 Introduction

Depression is a complex, multifactorial psychiatric disorder characterized by pervasive low mood, cognitive impairments, and disturbances in appetite and sleep, often leading to suicide [1]. With over 300 million affected globally, its prevalence continues to rise, posing an escalating public health crisis [2, 3]. The World Health Organization (WHO) has prognosticated that by 2030, depression will emerge as the leading global health burden [4]. Despite extensive research, significant gaps remain in understanding its etiology and underlying molecular mechanisms, hindering the development of rapid and effective treatments [5]. A deeper understanding of depression's pathophysiology is essential for discovering novel therapeutic strategies.

Recent evidence has increasingly implicated microglia, the resident immune cells of the central nervous system, in the pathogenesis of depression. Microglia play a vital role in maintaining neuronal health through synaptic pruning, a process crucial for brain development and plasticity. However, this process can become dysregulated in neuropsychiatric disorders, including depression [6, 7]. Specifically, excessive microglial pruning leads to synaptic deficits in brain regions involved in emotion regulation, such as the prefrontal cortex and hippocampus [8, 9]. Synaptic loss impairs neuronal communication, contributing to the cognitive and emotional disturbances that define depression [10, 11]. Postmortem studies have shown reduced synaptic density in the brains of depressed individuals, indicating the link between synaptic dysfunction and depression [12, 13]. Animal studies also support the therapeutic potential of modulating microglial activity, with inhibition of over‐reactive microglia or restoration of normal pruning alleviating depressive‐like behavior [6]. These findings underscore the promise of targeting microglial function as a strategy to treat depression.

Heat shock protein 60 (HSP60), a molecular chaperone primarily involved in protein folding and cellular stress responses, has a significant role in microglial function [14, 15]. Under stress or injury conditions, HSP60 expression in microglia adapts to environmental changes. It modulates microglial inflammatory responses via the toll‐like receptor 4 (TLR4) pathway, which is activated when HSP60 accumulates intracellularly [16, 17]. This activation triggers an inflammatory response, often contributing to neuronal damage in neurodegenerative diseases like Alzheimer's [18]. As a mitochondrial chaperone, HSP60 also prevents the accumulation of misfolded proteins, with its absence leading to cellular stress [19, 20]. This cellular stress may lead to an overactivation of microglia, potentially resulting in an exaggerated inflammatory response. In the context of depression, microglial activation and enhanced synaptic pruning, mediated by HSP60, may exacerbate synaptic damage and contribute to mood disorders and cognitive deficits. Despite these insights, the precise role of microglial HSP60 in depression remains unclear, and its potential as a therapeutic target warrants further investigation.

This study aims to explore how HSP60 deletion in microglia affects synaptic function and behavior. Using behavioral assays, immunofluorescence staining, and proteomics, we investigated the consequences of HSP60 deletion on microglial pruning and synaptic integrity. Additionally, microglia were depleted using the CSF1R inhibitor PLX3397, which alleviated depressive‐like behaviors and restored synaptic density. This work offers new insights into the role of HSP60 in microglial function and its implications for depression.

## 2 Methods

### 2.1 Animals

**FIGURE 1:**
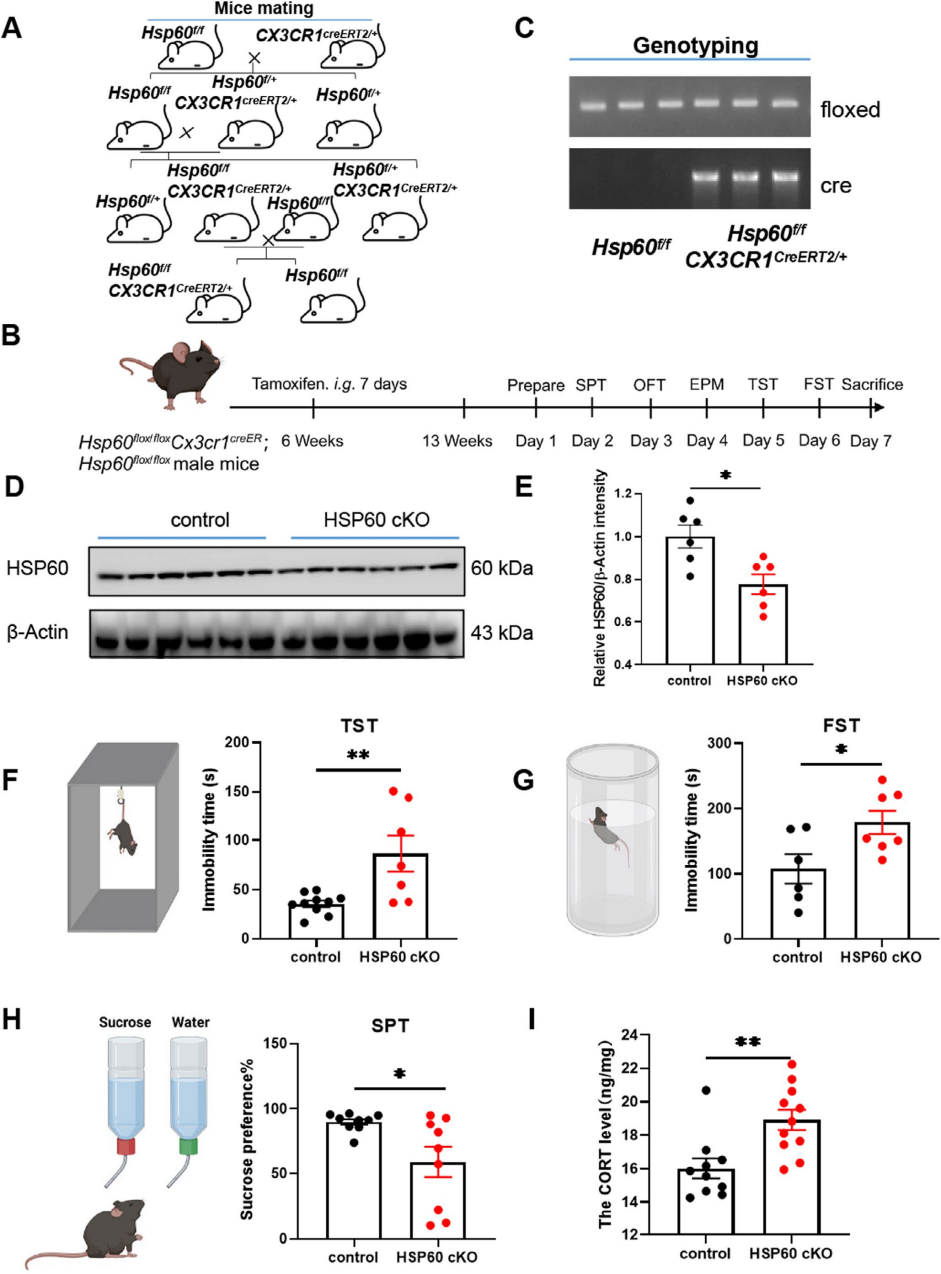
HSP60 deficiency of microglia induced depressive‐like behavior in male mice. (A) Transgenic mouse breeding scheme diagram. (B) Schematic diagram showing experimental procedures of the HSP60 cKO mice. (C) Genotyping of the *Hsp60* ^*f/f*^ mice and *Hsp60* ^*f/f*^ *CX3CR1* ^*CreER*^ mice. (D, E) The western blotting of the cortex in the control and HSP60 cKO mice. *n* = 6. (F–H) Tail suspension test (F), forced swimming test (G) and sucrose preference test (H) of control and HSP60 cKO mice. (I) The CORT level in the cortex of control and HSP60 cKO mice. *n* = 6–11. Mean ± SEM, Unpaired *t* test. ns, no significance; *p < 0.05; **p < 0.01; ***p < 0.001; ****p < 0.0001.

*CX3CR1* ^*CreER*^ mice (Stock No: 021160) were purchased from the Jackson Laboratory (Bar Harbor, USA), and *Hsp60* ^*flox/flox*^ mice (*Hsp60* ^*f/f*^) were gifted by Prof. Kunfu Ouyang (PKUSZ, China). The generation of the *Hsp60* ^*f/f*^ mouse has been described [21]. We obtained *Hsp60* ^*f/f*^ *CX3CR1* ^*CreER*^ mice by *Hsp60* ^*f/f*^ mice crossing *CX3CR1* ^*CreER*^ mice (Figure 1A). Mice were housed at 26°C ± 1°C under a 12‐h light/dark cycle with ad libitum access to standard rodent chow and water. All mice used were 13 weeks old. The experimental procedures were designed to minimize animal suffering. To induce microglial‐specific *Hsp60* gene deletion, 6‐week‐old *Hsp60* ^*f/f*^ *CX3CR1* ^*CreER*^ male mice were intragastrically injected with tamoxifen (TAM) (100 mg/kg/d) for 7 consecutive days and considered microglial‐specific *Hsp60* knockout (HSP60 cKO) mice (Figure 1B) [22]. Additionally, the littermate *Hsp60* ^f/f^ male mice were also treated with tamoxifen using the same procedure and were used as control mice. All procedures conformed to the guidelines set by the Institutional Animal Care and Use Committee (IACUC) of Peking University Shenzhen Graduate School (Approval No: AP0013004).

### 2.2 Mice Treatment

PLX3397 (Selleckchem Inc., Houston, TX) was formulated in a standard chow (1022, Beijing HFK Bioscience Co. Ltd., China). Groups of mice received PLX3397 (290 mg/kg of chow) for 21 days to eliminate microglia [23], and then were followed by withdrawal for 21 days prior to behavioral tests. Mice in control groups received the standard chow (1022, Beijing HFK Bioscience Co. Ltd., China).

### 2.3 Behavioral Assays

#### 2.3.1 Open Field Test

The open field test (OFT) was performed according to previously developed protocols [24]. Briefly, mice were habituated for 1 h before being placed in a 45 × 45 × 30 cm chamber for a 5‐min session. Locomotor activity, including total distance and time spent in the center, was recorded and analyzed using ANY‐Maze software.

#### 2.3.2 Tail Suspension Test

A tail suspension test (TST) was performed according to the reported protocols [25]. Briefly, mice were suspended by their tail for 5 min after a 1‐h habituation period. Immobility time was measured using an animal behavior observer (Noldus, Netherlands) and analyzed with EthoVision XT software.

#### 2.3.3 Forced Swimming Test

The forced swimming test (FST) was performed according to the reported protocols [26]. Mice were placed in a transparent cylindrical container (50 cm height, 20 cm diameter) filled with room‐temperature water. After 1 h of habituation, the animals were immersed for 5 min. Immobility duration was quantified when the mice remained motionless, only moving their heads to stay above the water. EthoVision XT software was used for analysis.

#### 2.3.4 Sucrose Preference Test

The sucrose preference test (SPT) was performed according to previously developed protocols [27]. Briefly, mice were habituated to two water bottles for 3 days, followed by a 20‐h water deprivation. Then, each mouse was given free access to one bottle of tap water and one of 1% sucrose solution for 5 h. Sucrose preference was calculated using the formula: 100% × sucrose intake (g)/[sucrose intake (g) + water intake (g)].

#### 2.3.5 Elevated Plus Maze

The Elevated Plus Maze (EPM) consisted of four arms (two open, two closed) elevated 1 m above the ground. Mice were placed at the center, facing an open arm, and allowed to explore for 5 min. Activity, including time spent in open, closed, and central areas, was recorded and analyzed via Noldus EthoVision XT10 software.

### 2.4 Western Blotting

According to the developed protocols, western blotting was performed [28]. Briefly, denatured cortex samples (boiled at 100°C for 10 min) were separated on SDS‐PAGE gel and then transferred to the nitrocellulose membrane (250 mA, 3 h). The membrane was blocked with 5% nonfat milk or Bovine Serum Albumin (BSA) in TBST solution, then incubated in primary antibody (list of antibodies with dilution used, see Table 1), overnight at 4°C. The next day, the membrane was washed with TBST solution for 4 times (10 min every time), the membrane was then treated with a secondary antibody for 1 h at room temperature, and membrane was washed with TBST solution for 4 times (20 min every time) again. Chemiluminescence detection was performed using an ECL SuperSignal kit (4A Biotech, Beijing, China) and developed on an e‐blot system (Touch Imager XLI, Shanghai, China). Band intensity was quantified using ImageJ software.

**TABLE 1:** Detailed antibody information.

Antibody name	Company	Cat number	Dilute
HSP60	Abcam	ab190828	1/1000
CD68	Abcam	ab283654	1/1000
CD206	Cell signaling technology	24,595	1/1000
Arg1	Cell signaling technology	93,668 T	1/1000
NR2A	Santa Cruz	SC‐1468	1/500
NR2B	Santa Cruz	SC‐1469	1/500
NR2C	Abcam	ab182277	1/1000
NR2D	Abconal	A10080	1/1000
AMPAR1	Cell signaling technology	13,185	1/1000
AMPAR2	Cell signaling technology	13,607	1/1000
DrebrinA	Cell signaling technology	12,243	1/1000
Synaptophysin	Abcam	ab32127	1/1000
Snap25	Cell signaling technology	5308	1/1000
Synapsin‐1	Cell signaling technology	5297	1/1000
PSD95	Abcam	ab18258	1/1000
β‐Actin	Santa Cruz	SC‐47778	1/500

### 2.5 Enzyme Linked Immunosorbent Assay

Enzyme linked immunosorbent assay (ELISA) was performed as previously reported [29]. Briefly, frozen brain samples were homogenized in RIPA buffer. According to the manufacturer's protocols, the expression of neurotransmitter was quantified using ELISA kit (mlbio, Shanghai, China, CORT: ml037564; Glutamate: ml616547V; GABA: mI001894V). 50 μL standard/sample and 100 μL HRP‐labeled antibody solutions were incubated for 1 h at 37°C, followed by four washes (1 min every time). 50 μL of solution A and 50 μL of solution B were added for 15–20 min at 37°C, and the reaction was stopped with a stop solution. Then the optical density was measured by microplate reader (BioTek, USA).

### 2.6 Golgi Staining

According to the developed protocols, Golgi staining was performed [30]. Golgi staining was performed using the FD Rapid Golgi Stain Kit (FD, Neuro Technologies, Ellicott City, MD). Brains were rinsed in double‐distilled water, immersed in impregnation solutions (A/B), and stored at room temperature for 2 weeks. Tissues were then transferred to solution C for 72 h, followed by freezing. Sections (150 μm) were prepared and mounted on gelatine‐coated slides. Dendritic spines were quantified by examining high‐resolution images (100× magnification) under an inverted fluorescence microscope (Olympus IX73). Spine counts were performed manually along secondary and tertiary dendrites within a 10 μm segment from at least 10 neurons per sample, ensuring coverage across various cortical layers.

### 2.7 Quantitative Real‐Time PCR

Total RNA was extracted from mouse cortical tissue using the TransZol Up Plus Kit (ER501‐01, TransGen Biotech, Beijing, China). cDNA synthesis was performed with the TransScript One‐Step gDNA Removal and cDNA Synthesis SuperMix (AT311‐04, TransGen Biotech, Beijing, China) according to the manufacturer's instructions. qRT‐PCR was conducted using TransStart Top Green qPCR SuperMix (AQ131‐03, TransGen Biotech, Beijing, China). Primer sequences are listed in Table 2, and primers were synthesized and purified by Beijing Qingke Biotechnology.

**TABLE 2:** The sequence of the primers.

Primer	Sequence (5′–3′)
mertk‐F	TTAATACCTCTGCTTCGCCACATCTG
mertk‐R	GACCAGCCAATCTCATTCCGACAG
cd18‐F	AGCAGAAGGACGGAAGGAACATTTAC
cd18‐R	ATGACCAGGAGGAGGACACCAATC
adgrb1‐F	CTGCCTGAAGAGGAGAAGATGAAGC
adgrb1‐R	AGGGTGAGTGAGTGATTAGGGAAGTC
sirpa‐F	CCTACAACATCTCCAGCACAGTCAG
sirpa‐R	TCGGATGAAGTTAGACAGGTTAGCAATC
vtn‐F	TGAGCTAGATGAGACGGCAGTGAG
vtn‐R	CCCTGACAGTTGATGCGAGTGAAG
c3‐F	CCAGCTCCCCATTAGCTCTG
c3‐R	GCACTTGCCTCTTTAGGAAGTC
gpr34‐F	CTCAGGAGTGCCAAATGTCAC
gpr34‐R	GCCCAGAAATACATAGAGGGCAA
tyrobp‐F	GAGTGACACTTTCCCAAGATGC
tyrobp‐R	CCTTGACCTCGGGAGACCA
cybb‐F	TGTGGTTGGGGCTGAATGTC
cybb‐R	CTGAGAAAGGAGAGCAGATTTCG
trem2‐F	CTGGAACCGTCACCATCACTC
trem2‐R	CGAAACTCGATGACTCCTCGG
p2y6‐F	AGCAAGGCGGCTCGTATG
p2y6‐R	TCTCCAGCACAGGGCAAGA
cd206‐F	CAGGTGTGGGCTCAGGTAGT
cd206‐R	TGTGGTGAGCTGAAAGGTGA
gapdh F	CAGTGCCAGCCTCGTCCCGTAGA
gapdh R	CTGCAAATGGCAGCCCTGGTGAC

### 2.8 Immunofluorescence

Brain tissue sections (30 μm thick) were cut by frozen slicer (CM1860UV, Leica, Germany), followed by three washes in PBS (10 min each). Sections were blocked with 10% goat serum and 0.3% Triton X‐100 in PBS for 1 h at room temperature. After blocking, primary antibodies (Iba‐1: Wako, 019‐19741, 1:500; CD86: Novus, NBP2‐25208, 1:200; PSD95: Thermo, MA1‐045, 1:500; Vglut1: SySy, 135,304, 1:1000; Gad65: Merck, ZRB1091, 1:100) were applied overnight at 4°C. The following day, sections were washed 3 times (10 min every time) and incubated with secondary antibodies for 1 h at room temperature. After washing 3 times, slices were mounted and imaged with a Nikon A1R confocal microscope. Marker intensity was quantified using ImageJ software (NIH).

### 2.9 Microglial Morphology Analysis

The quantitative analysis of microglia morphological changes in a biological system was performed using ImageJ software (NIH) along with the Analyze Skeleton (2D/3D) plugin [31]. Microglia were immunolabeled with Iba‐1 staining, which highlighted the contours and skeletons of the cell bodies. In summary, the photomicrographs were first converted into 8‐bit images, followed by the application of a FFT bandpass filter. The brightness and contrast were then adjusted to optimize the visibility of microglial branches. To enhance image contrast, the unsharp mask was applied, and any resulting noise was reduced using the despeckle function. Next, the image was binarized, and additional despeckle, close, and remove outliers functions were applied to eliminate single‐pixel background noise and fill gaps between the processes. The image was subsequently skeletonized and further analyzed using the Analyze Skeleton (2D/3D) function.

### 2.10 Analysis of 3D Microglia Engulfment

The method used to analyze microglial morphology was precisely as we previously described [32, 33]. The z‐stack images (at 1 μm intervals) were captured using a Zeiss LSM980 confocal microscope with a 63× lens. Microglia were selected randomly based on positive staining for IBA1, ensuring an unbiased approach. Individual microglia, including those with PSD95/Vglut1/Gad65 signaling, were isolated using Image J and transferred to Imaris 9.0.0 software (Oxford Instruments, USA). Initially, 3D volume surface renderings of the microglial channels were generated to compute each microglial cell's volume and surface area. Subsequently, a new channel representing engulfed PSD95/Vglut1/Gad65 was established using the mask function to determine the volume of engulfed PSD95/Vglut1/Gad65. The engulfment percentage was calculated as the volume of internalized PSD95/Vglut1/Gad65 puncta divided by the volume of the microglial cell.

### 2.11 Sample Preparation and NanoLC–MS/MS Analysis

Proteins were extracted using RIPA buffer (1% Triton X‐100, 1% deoxycholate, 0.1% SDS) with protease inhibitors (Roche, 05892970001, Basel, Switzerland), followed by homogenization and centrifugation. Protein concentration was determined with the Pierce BCA Protein Assay Kit (Thermo Fisher Scientific, 23,227, MA, USA).

For digestion, 100 μg of protein was reduced with 10 mM dithiothreitol (DTT, Sigma‐Aldrich, St. Louis, MO) at 70°C for 1 h, alkylated with 50 mM iodoacetamide (IAA, Sigma‐Aldrich) at room temperature for 15 min, and desalted using 0.5 M ammonium bicarbonate. Proteins were digested overnight with trypsin (Promega, V5111, Madison, WI) at a 1:50 ratio at 37°C, then lyophilized and stored at −80°C.

For Nano LC–MS/MS, peptides were reconstituted in 30 μL of 0.1% formic acid and injected onto an UltiMate 3000 RSLC nano system (Thermo Fisher Scientific) with a C18 precolumn (Acclaim PepMap 100 C18, 100 μm × 20 mm, 3 μm) and a C18 tip column (Acclaim PepMap RSLC, 75 μm × 250 mm, 2 μm). The gradient used mobile phases A (0.1% formic acid) and B (80% acetonitrile in 0.1% formic acid) from 5% to 95% B over 90 min, at a flow rate of 300 nL/min. The column was coupled to a Q Exactive Plus mass spectrometer (Thermo Fisher Scientific), with MS1 scans (300–1500 m/z) at 70,000 resolution and MS2 spectra at 17,500 resolution, using dynamic exclusion for 30 s.

Protein identification and quantification were performed using Proteome Discoverer 2.4 with Sequest HT. Differentially expressed proteins were identified with a 1.5‐fold change cutoff and *p* < 0.05. Gene Ontology (GO) annotation and KEGG (Kyoto Encyclopedia of Genes and Genomes) pathway analysis were performed using the OmicsBean database, and protein–protein interaction (PPI) networks were analyzed using the OmicsBean web tool.

### 2.12 Statistical Analysis

All data were analyzed by GraphPad Prism 8.0 software. Data were presented as Mean ± SEM. Unpaired *t* test were performed to compare different groups. *p* < 0.05 was regarded as significant. ns, no significance; *p < 0.05; **p < 0.01; ***p < 0.001; ****p < 0.0001.

## 3 Results

### 3.1 HSP60 Deficiency of Microglia Induced Depressive‐Like Behavior in Male Mice

The HSP60 knockout in microglia of the mice was generated using CX3CR1‐CreER to specifically delete HSP60 in microglia through tamoxifen treatment at 6 weeks of the male mice (Figure 1A,B) and validated HSP60 deletion in brain tissues through genotyping and western blotting (Figure 1C–E).

Learned helplessness and anhedonia are two main core manifestations of depressive disorder, with the TST, FST, and SPT serving as widely employed paradigms to assess the behavioral phenotypes associated with these conditions, respectively [34]. In TST and FST, the immobility time in HSP60 cKO mice was significantly increased compared with the control mice (*p* < 0.01; *p* < 0.05; Figure 1F,G). In the SPT, the sucrose preference ratio was significantly lowered in HSP60 cKO mice, suggesting depressive‐like behavior (*p* < 0.05; Figure 1H). Additionally, the results of the ELISA revealed an increase of cortical corticosterone (CORT) levels in HSP60 cKO mice, indicating activation of the hypothalamic–pituitary–adrenal (HPA) axis, which may reflect a heightened stress response or an altered physiological state in these mice (*p* < 0.05; Figure 1I). Together, these findings provided further evidence of a depressive‐like phenotype in HSP60 cKO mice.

### 3.2 Locomotion and Anxiety Level Did Not Change in HSP60 cKO Male Mice

**FIGURE 2:**
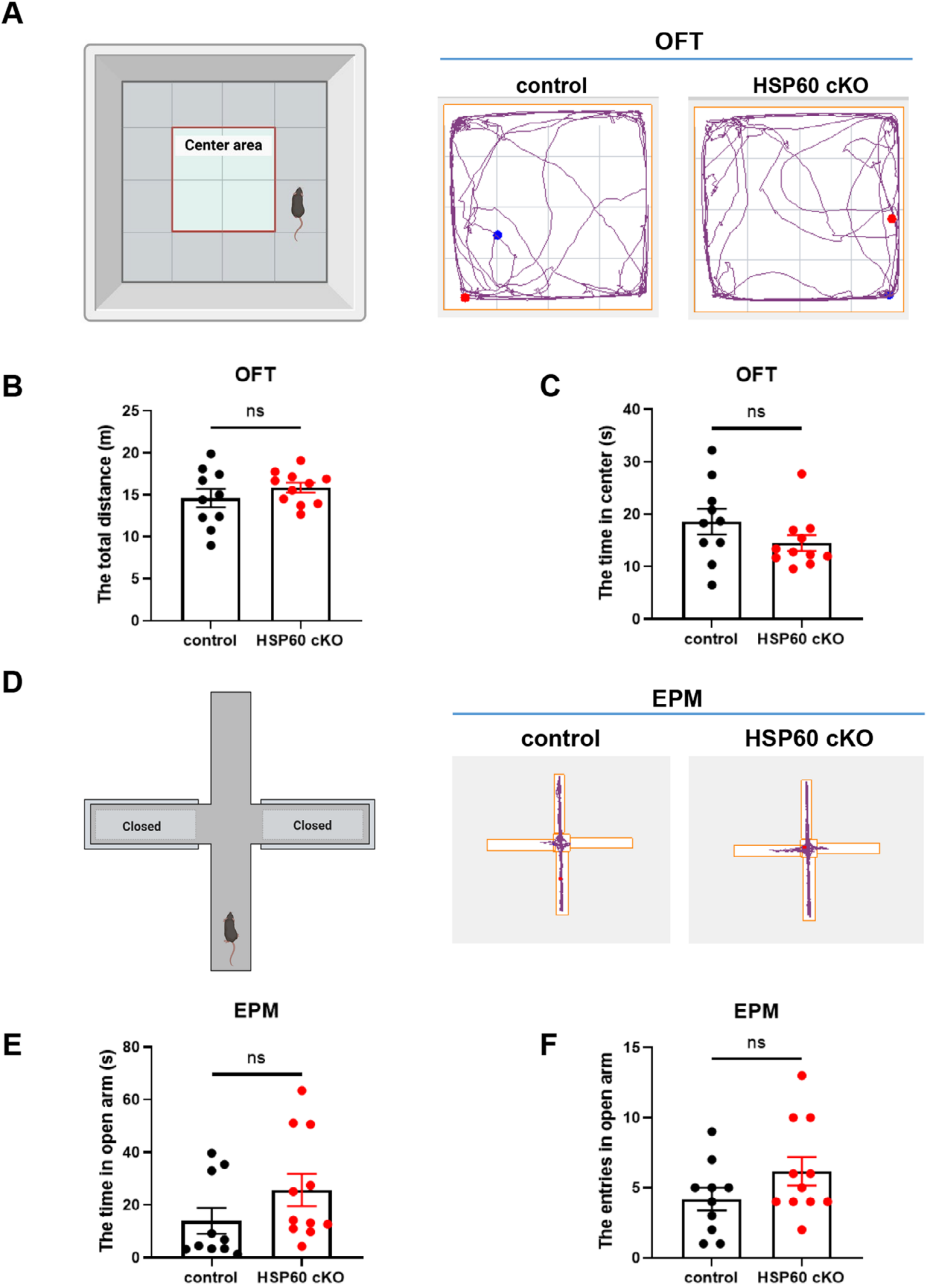
Locomotion and Anxiety Level did not Change in HSP60 cKO Mice. (A) Representative images of movement trace in OFT. (B, C) The total distance (B) and center time (C) in OFT of control and HSP60 cKO mice. (D) Representative images of movement trace in EPM. (E, F) The time (E) and entries (F) in the open arm of control and HSP60 cKO mice. Control: *N* = 10; HSP60 cKO: *N* = 11. Mean ± SEM, Unpaired *t* test. ns, no significance; *p < 0.05; **p < 0.01; ***p < 0.001; ****p < 0.0001.

Depressive and anxiety disorders are frequently comorbid, but the nature of their relationship remains a subject of ongoing debate. In the TST and FST, increased immobility time is typically interpreted as a marker of depression, though it may also reflect reduced spontaneous movement. To distinguish whether the prolonged immobility observed in HSP60 cKO mice was primarily due to depressive symptoms rather than impaired locomotor activity, we conducted the OFT (Figure 2A). As expected, no significant changes were found in the total distance traveled between the HSP60 cKO and control mice (*p* > 0.05; Figure 2B). The time that the HSP60 cKO and control mice spent in the middle area, usually reflecting anxiety‐like behavior, did not alter either (*p* > 0.05; Figure 2C). In addition, we examined whether the HSP60 cKO mice were accompanied by anxiety‐like behavior in the EPM (Figure 2D). Of interest, there was no profound change in the time spent and entries in the open arms between the HSP60 cKO and control mice (*p* > 0.05; Figure 2E).

### 3.3 The Deletion of HSP60 Activated the Pruning Ability of Microglia in the Mouse Cortex

**FIGURE 3:**
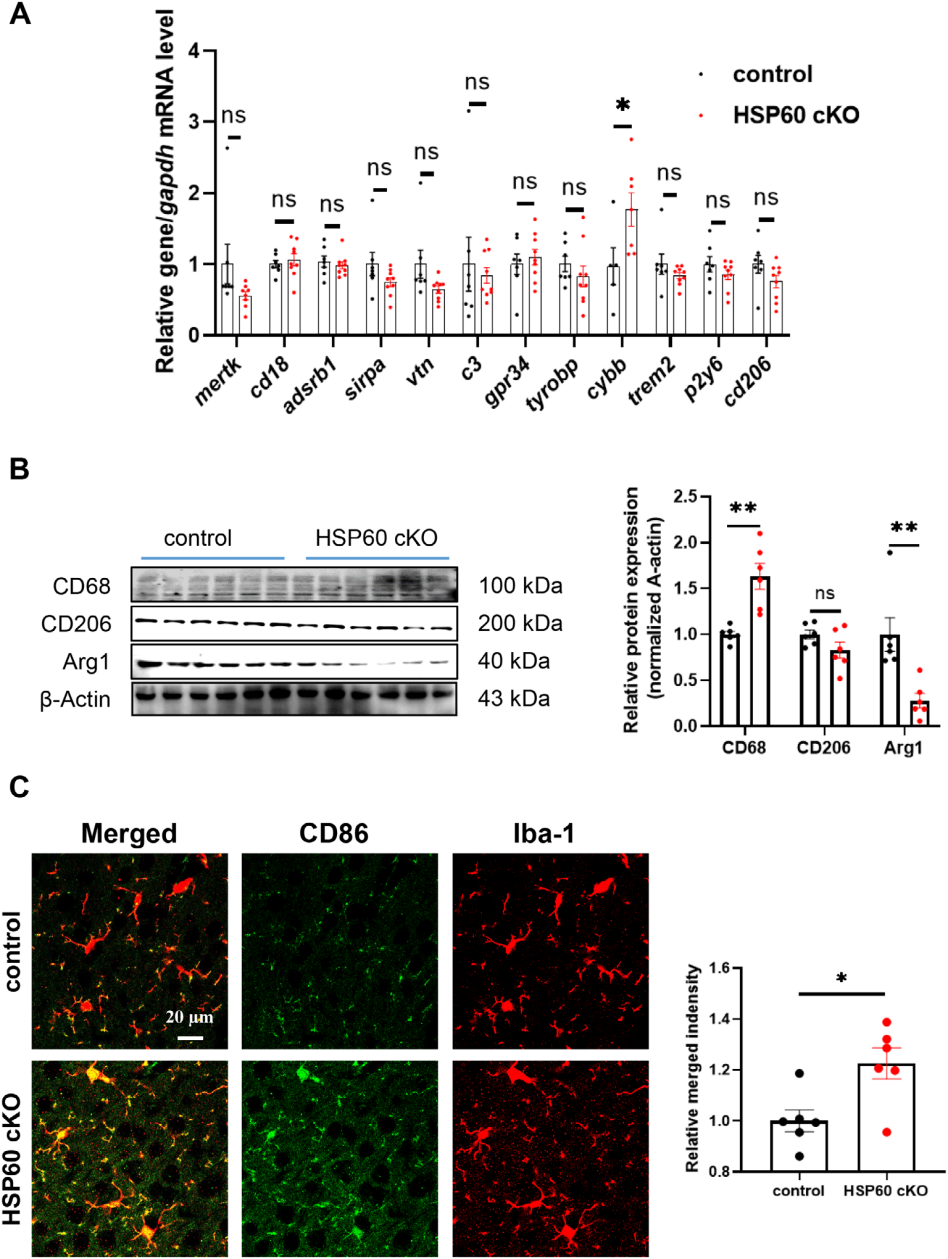
The deletion of HSP60 activated microglia in the mouse cortex. (A) The transcriptional level of *merk*, *cd18*, *adsrb1*, *sirpa*, *vtn*, *c3*, *gpr34*, *tyrobp*, *cybb*, *trem2*, *p2y6*, *cd206* of the cortex. (B) The expression level of CD68, CD206, Arg1 in the mouse cortex. (C) The expression level of CD86 in microglia of the control and HSP60 cKO mice. Scale bar: 20 μm. *n* = 6. Mean ± SEM, Unpaired *t* test. ns, no significance; *p < 0.05; **p < 0.01; ***p < 0.001; ****p < 0.0001.

Previous studies have established that excessive microglial activation is closely associated with the pathophysiology of depression, with heightened inflammatory responses in the brain contributing to the manifestation of depressive‐like symptoms [6, 35]. In line with these findings, our qPCR analysis of the cortex revealed a significant upregulation of *cybb* transcription, which encodes the NADPH oxidase 2, while the results of western blotting demonstrated increased CD68 levels and decreased Arg1 expression in HSP60 cKO mice (*p* < 0.05; *p* < 0.01; Figure 3A,B). These molecular alterations further suggest the presence of an inflammatory microenvironment. Additionally, immunofluorescence analysis showed elevated expression of CD86 in Iba‐1‐positive cells (microglia), reinforcing the idea of microglial activation (*p* < 0.05; Figure 3C).

**FIGURE 4:**
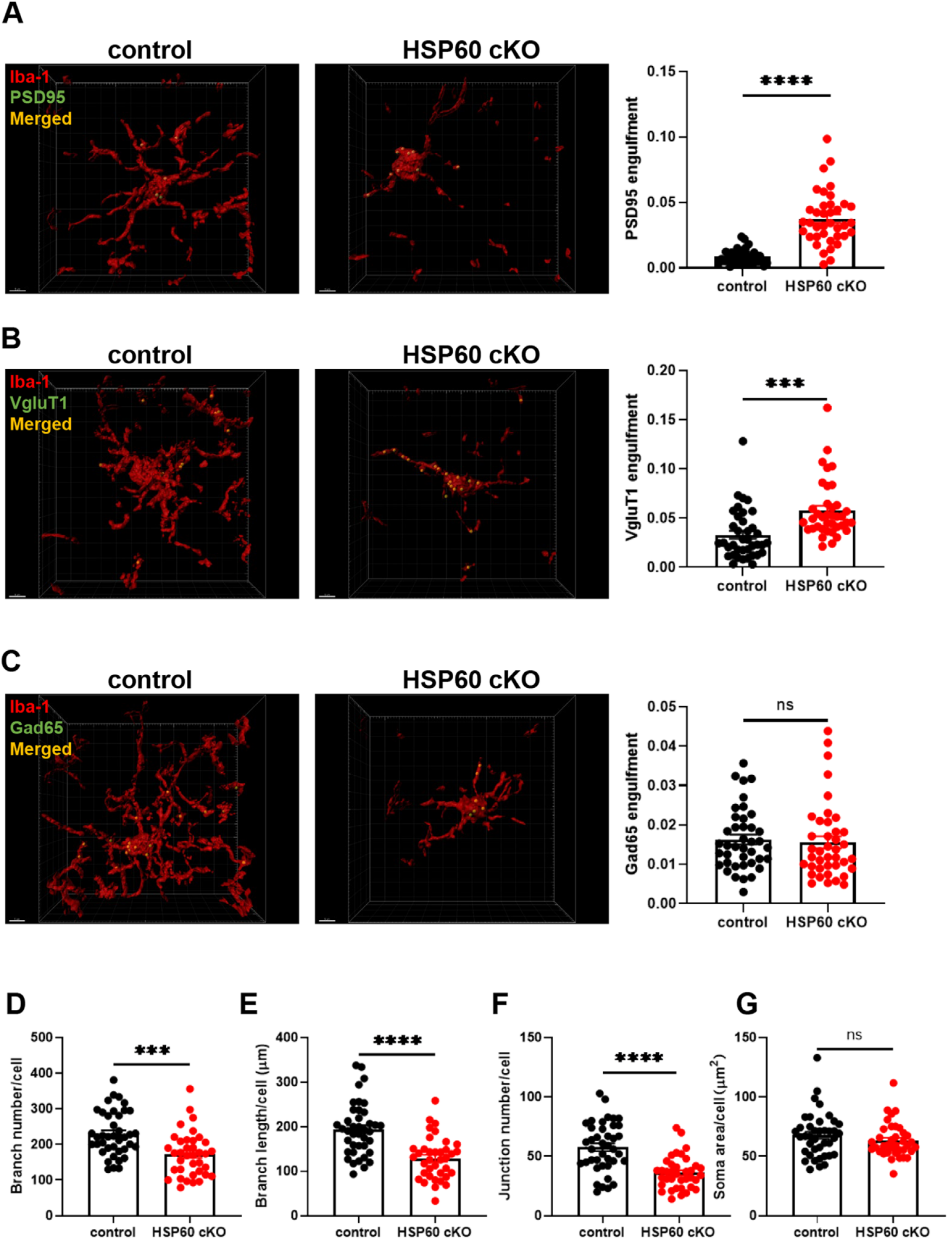
The deficiency of HSP60 activated the pruning ability of microglia in the mouse cortex. (A–C) PSD95 (A), Vglut1 (B) and Gad65 (C) engulfment of the microglia in the control and HSP60 cKO mice. Scale bar: 5 μm. (D–G) Morphology of microglia in the control and HSP60 cKO mice. Branch number (D), Branch length (E), Junction number (F), Soma perimeter (G). *n* = 6. Mean ± SEM, Unpaired *t* test. ns, no significance; *p < 0.05; **p < 0.01; ***p < 0.001; ****p < 0.0001.

Subsequently, we further investigated microglial phagocytic activity using immunofluorescence co‐staining and analysis with Imaris software. The results revealed that, following HSP60 knockout, microglia exhibited an increased phagocytosis of PSD95 and Vglut1, suggesting that microglia may be actively involved in the removal of these synaptic components (*p* < 0.0001; *p* < 0.001; Figure 4A,B). However, no significant change was observed in the phagocytosis of Gad65, a marker of inhibitory synapses, indicating a selective alteration in microglial synaptic pruning (*p* > 0.05; Figure 4C).

Microglia, as the resident immune cells of the central nervous system, undergo significant morphological and functional changes in response to activation, with these alterations closely associated with their role in neuroinflammation [36]. Keeping with the observed inflammatory changes, the knockout of HSP60 in mice resulted in notable morphological alterations of microglial cells. Specifically, microglia in HSP60 cKO mice exhibited decreased complexity in their morphology, with a reduced number of branches, fewer junctions, and shorter branch lengths (*p* < 0.001; *p* < 0.0001; *p* < 0.0001; Figure 4D–F). Interestingly, the soma size of the microglia did not show significant changes, suggesting that while the microglial network became less elaborate, the size of the soma remained unaffected (*p* > 0.05; Figure 4G).

### 3.4 The Deletion of HSP60 Caused Synaptic Deficits and Modified Relative Signaling Pathways in Mouse Cortex

**FIGURE 5:**
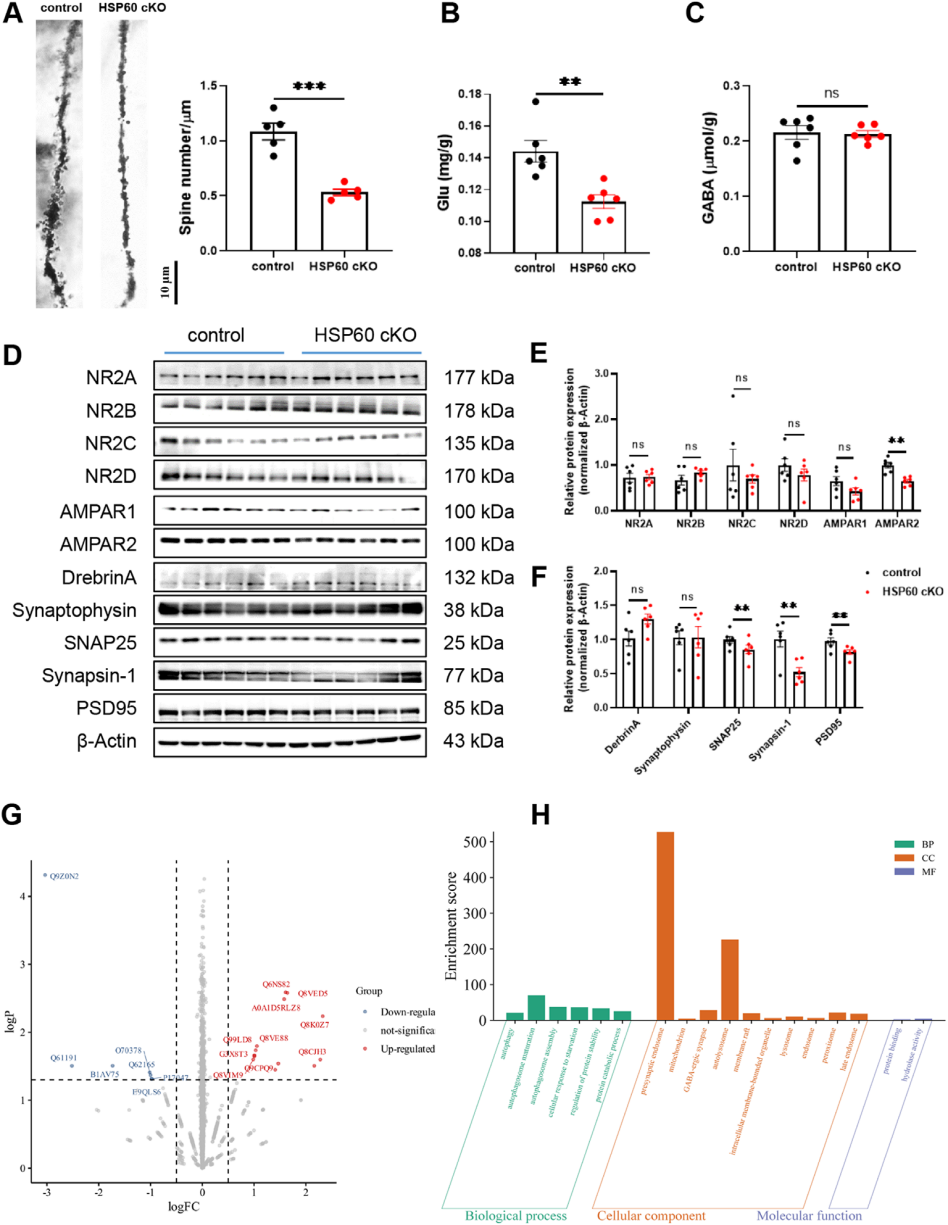
The deletion of HSP60 caused synaptic deficits and modified relative signaling pathways in the mouse cortex. (A) Spine density and column graph of Golgi staining in the mouse cortex. *n* = 5. (B, C) The Glu (B), GABA (C) level in the cortex of control and HSP60 cKO mice. (D–F) Representative western blotting images of NR2A, NR2B, NR2C, NR2D, AMPAR1, AMPAR2, DrebrinA, Synaptophysin, SNAP25, Synapsin‐1, PSD95 and their bar graph in the mouse cortex. (G) The Volcano plots of significantly different proteins in the cortex of control mice and HSP60 cKO mice. (H) GO enrichment analysis of significantly different proteins in the cortex of control and HSP60 cKO mice. *n* = 6. Mean ± SEM, Unpaired *t* test. ns, no significance; *p < 0.05; **p < 0.01; ***p < 0.001; ****p < 0.0001.

Given the evidence of dysregulated synaptic pruning, Golgi staining further revealed a significant reduction in synapse density in the cortex of HSP60 cKO mice (*p* < 0.001; Figure 5A). In addition, ELISA analysis showed a marked decrease in glutamate (Glu) levels, while GABA levels remained unchanged, suggesting a selective reduction in excitatory neurotransmission without a concomitant alteration in inhibitory neurotransmission (*p* < 0.01; *p* > 0.05; Figure 5B,C).

Consistent with the reduced synapse density and synaptic dysfunction, western blotting analysis revealed a significant downregulation of AMPAR2, Synapsin‐1, SNAP25 and PSD95 expression in the cortex of HSP60 cKO mice (*p* < 0.01; Figure 5D–F). These proteins are key markers of excitatory synapses and synaptic function, and their decreased expression further supports the notion of synaptic dysfunction in these mice (Figure 5D–F).

To elucidate the molecular mechanism of microglial HSP60, we first sought to identify significantly changed signaling pathways between control mice and HSP60 cKO mice. With the screening criteria set at *p* value < 0.05 and | log2FC | > 0.583, we further found 20 significant differentially expressed proteins (DEPs) among which 13 proteins were up‐regulated and 7 proteins were down‐regulated in the HSP60 cKO mice (volcano plot; Figure 5G). Gene Ontology (GO) analysis from 20 DEPs showed that these genes are associated with the presynaptic endosome (Figure 5H), which was reported to play a crucial role in maintaining synaptic function, ensuring efficient neurotransmitter release and supporting synaptic repair and plasticity [37].

### 3.5 PLX3397 Improved Depressive‐Like Behavior in HSP60 cKO Male Mice

**FIGURE 6:**
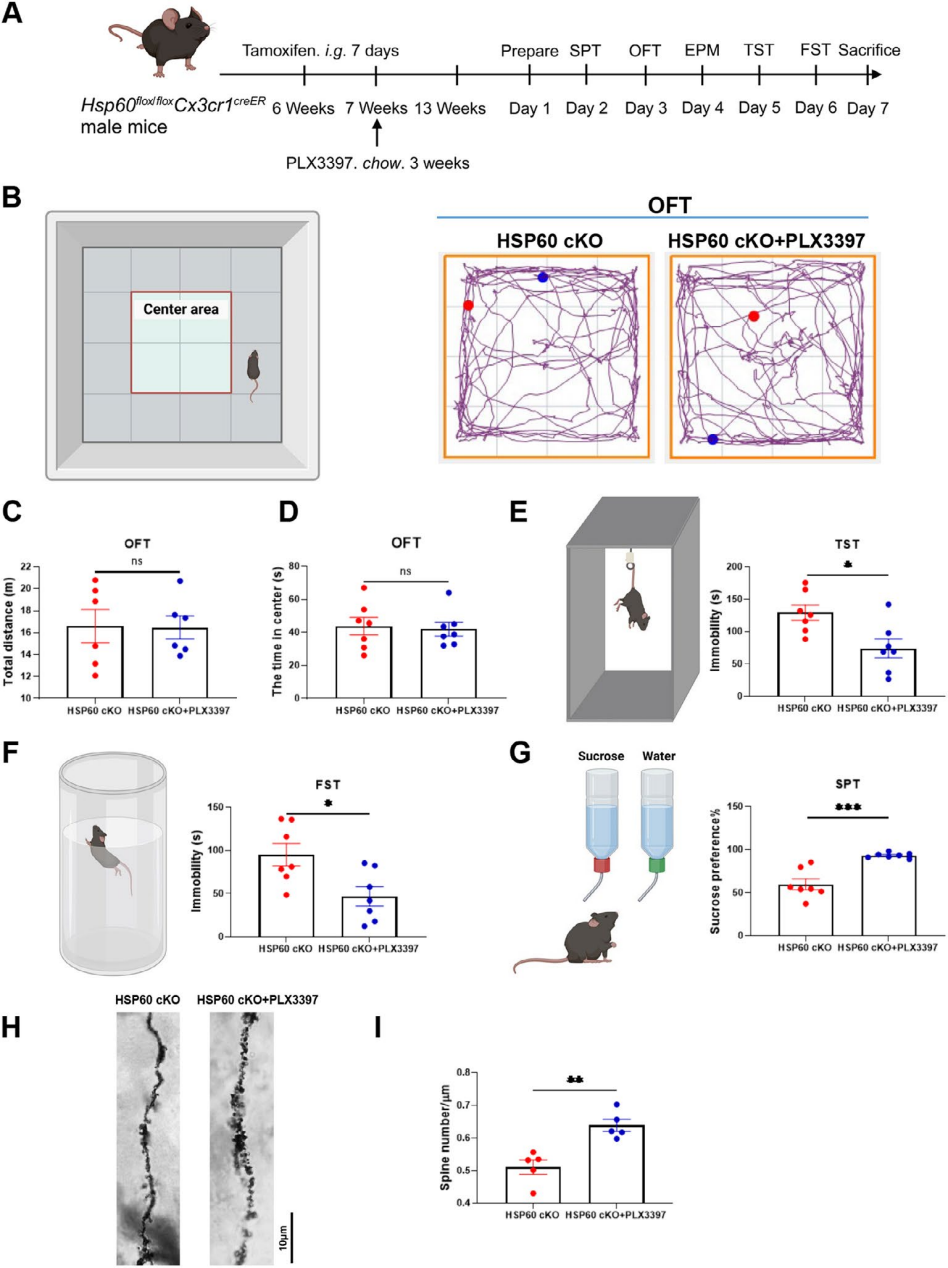
PLX3397 improved depressive‐like behavior in HSP60 cKO mice. (A) Timeline of PLX3397 administration and behavioral tests of HSP60 cKO male mice. (B) Representative images of movement trace in OFT. (C, D) The total distance and center time in OFT of the mice. (E–G) Tail suspension test, forced swimming test, and sucrose preference test of the mice. (H, I) Spine density and column graph of Golgi staining in mouse cortex (*n* = 5). *n* = 7. Mean ± SEM, Unpaired *t* test. ns, no significance; *p < 0.05; **p < 0.01; ***p < 0.001; ****p < 0.0001.

To further investigate the role of microglial activation in the depressive‐like phenotype of HSP60 cKO mice, we treated a subset of animals with PLX3397, a selective CSF1R inhibitor that could deplete microglia (Figure 6A). To rule out potential confounding effects of PLX3397 on motor activity, we conducted an open field test (Figure 6B). The results showed no significant differences in total distance traveled or time spent in the center, indicating that PLX3397 did not affect locomotion or anxiety levels (*p* > 0.05; Figure 6C,D). This ensured that the behavioral effects observed in subsequent tests could be attributed to the drug's impact on microglial depletion. In the TST and FST, PLX3397 administration significantly reduced immobility time, suggesting an antidepressant‐like effect (*p* < 0.05; *p* < 0.05; Figure 6E,F). Additionally, PLX3397 treatment increased sucrose preference in the SPT, further indicating a reduction in anhedonia (*p* < 0.001; Figure 6G). Importantly, these behavioral improvements occurred without any changes in locomotion or anxiety‐like behavior, confirming that the antidepressant effects of PLX3397 are not confounded by motor behavior. Consistent with these behavioral results, Golgi staining revealed an increase in synapse density in the cortex of PLX3397‐treated HSP60 cKO mice, suggesting that the antidepressant‐like effects of PLX3397 may be linked to enhanced synaptic remodeling or preservation, potentially through microglial modulation (*p* < 0.01; Figure 6H,I).

## 4 Discussion

This study elucidated the role of HSP60 in regulating depressive‐like behaviors in male mice. Our results found that the deficiency of HSP60 in microglia may lead to synaptic defects by promoting excessive pruning of excitatory synapses, which contributes to the depressive‐like phenotype. Interestingly, HSP60 knockout activated microglia and enhanced their phagocytic function. Notably, treatment with PLX3397 effectively reversed the depressive‐like symptoms and improved synaptic function in HSP60 cKO male mice. These findings underscore the critical role of HSP60 in microglia for maintaining neuronal health.

Microglia, the primary immune cells in the central nervous system, are closely linked to neuroinflammation and synaptic pruning. Previous studies have shown that abnormal activation of microglia and neuroinflammation are closely associated with the development of depression [6, 7]. HSP60, as a molecular chaperone, plays a crucial role in protein folding and quality control within cells, and is also involved in immune responses. Studies have highlighted its involvement in microglial inflammatory responses, particularly through the TLR pathway [16, 38]. HSP60 acts as an endogenous immunostimulatory factor, activating microglia via TLR4, leading to inflammation [16]. Moreover, one study also found that HSP60 could regulate IL‐1β‐induced microglia inflammation by activating the p38 MAPK pathway, further confirming the mechanism of action of HSP60 in neuroinflammation [39]. This mechanism is significant in neurodegenerative diseases like Alzheimer's, where excessive microglial activation contributes to neuronal damage. Nevertheless, as a mitochondrial chaperone, the absence of HSP60 leads to protein misfolding in mitochondria, inducing stress responses, mitochondrial dysfunction, and causing chronic inflammation [19, 20], which could result in the activation of microglia. This activation can increase synaptic pruning, potentially causing synaptic damage, leading to mood disorders. In expectation, HSP60 deficiency in microglia induced depressive‐like behavior in the mice. Briefly, while the accumulation of HSP60 promotes inflammation through the TLR4 pathway in some contexts, its absence causes a different type of dysfunction, leading to increased microglial activation due to mitochondrial stress.

Concurrently, the results of ELISA indicated an increase in CORT levels in the cortex of HSP60 cKO mice. CORT is strongly linked to depressive‐like behaviors through its role in the stress response of the organism. Chronic elevation of CORT levels, often resulting from prolonged or excessive stress, can lead to structural and functional changes in the brain, particularly in regions involved in mood regulation, such as the hippocampus and prefrontal cortex [40]. These changes may include neuronal atrophy, impaired neuroplasticity, and disruption of neurogenesis, all of which contribute to the development of depressive symptoms. Furthermore, elevated CORT levels can activate neuroinflammatory pathways, exacerbate synaptic pruning, and impair cognitive functions, which are commonly observed in individuals with depression [41, 42].

As previously mentioned, the loss of HSP60 leads to mitochondrial dysfunction then results in the generation of reactive oxygen species (ROS), which triggers the activation of microglia. Our findings indicated that the transcriptional level of *cybb* (the gene encoding NADPH oxidase 2, NOX2) was elevated in the cortex, suggesting that microglial activation and the associated oxidative stress are involved in the observed pathological changes, which likely reflect an increase in ROS production [43]. Additionally, the western blotting results showed an increase in CD68 expression, a decrease in Arg1 expression, and no significant change in CD206 levels, suggesting that the knockout of HSP60 may lead to an activation of microglia, as evidenced by the elevated expression level of CD68, which labels the lysosome and is a marker for microglial activation and phagocytic activity [44, 45]. The decrease in Arg1 expression, typically associated with the anti‐inflammatory role of microglia, indicates a shift towards a more pro‐inflammatory state, which further supports the idea of an altered inflammatory response in the context of HSP60 knockout [46]. Furthermore, the results of the immunofluorescence assay indicated an increase in CD86 expression in Iba‐1 positive cells, indicating heightened microglial activation. CD86 is a co‐stimulatory molecule associated with pro‐inflammatory responses, and its upregulation in microglia indicates a shift towards a more inflammatory phenotype [47, 48]. These findings support the idea that the loss of HSP60 triggers microglial activation and may contribute to neuroinflammation, which may play a role in the development of depressive‐like behaviors in the HSP60 cKO mice.

Considering that microglial activation is closely related to changes in microglial morphology, we performed immunofluorescence staining followed by analysis using Imaris software [49]. The results revealed that HSP60 knockout led to less complex microglial morphology, characterized by the increase in the number of branches and junctions, and longer branches. Microglia, as the resident macrophages of the brain, play a key role in phagocytosis and synaptic pruning [49]. Consistently, our results showed that HSP60 knockout enhanced the phagocytic ability of microglia for PSD95 and Vglut1, both markers of excitatory synapses, while their phagocytic capacity for Gad65, a marker of inhibitory synapses, remained unchanged, indicating that HSP60 knockout may selectively affect the pruning of excitatory synapses.

As expected, the results of Golgi staining also indicated a reduction in synapse number, further supporting the idea that HSP60 knockout leads to synaptic pruning. Additionally, ELISA results showed a decrease in Glu levels in the cortex, with no change in GABA levels, suggesting a reduction in excitatory neurotransmission without a corresponding change in inhibitory neurotransmission [50]. This could indicate a disruption in the excitatory–inhibitory balance, with a decrease in glutamate potentially reflecting impaired synaptic transmission or loss of excitatory synapses, further supporting the idea that HSP60 knockout may lead to a shift in the balance of neurotransmission, which could contribute to the development of depressive‐like behaviors.

Moreover, the western blotting results showed a decrease in the expression of AMPAR2, synapsin‐1, and PSD95, while the levels of NR2A, NR2B, NR2C, NR2D, AMPAR1, DrebrinA and synaptophysin did not show significant changes. AMPAR2 is one of the subunits of the AMPA‐type glutamate receptor, primarily responsible for postsynaptic depolarization and fast synaptic transmission [51]. Its downregulation may lead to weakened synaptic transmission, thereby affecting the function of neural networks, particularly synaptic plasticity. Synapsin‐1 plays a crucial role in the storage, transport, and release of synaptic vesicles [52]. Its downregulation suggests that the storage and release functions of synaptic vesicles may be impaired, leading to reduced neurotransmitter release efficiency, which in turn affects excitatory synaptic transmission. PSD95 is a postsynaptic density protein involved in stabilizing the postsynaptic structure, especially the anchoring of AMPA receptors [53]. Its downregulation may lead to the disruption of postsynaptic density, which in turn affects AMPA receptor function and synaptic plasticity, further influencing the stability of neural networks. Overall, the reduction in AMPAR2, SNAP25, synapsin‐1, and PSD95 suggested a potential loss of excitatory synaptic components and impaired synaptic plasticity. Moreover, the results of mass spectrum showed that the DEPs were associated with presynaptic endosome by GO enrichment analysis, which supported that knockout of microglial HSP60 could result in synaptic deficiency.

Building on our prior findings that demonstrated HSP60 knockout leads to excessive microglial pruning of excitatory synapses, we further investigated whether modulating microglial activity could mitigate the resulting depressive‐like behaviors. Recognizing that microglial overactivation contributes to synaptic loss and neuroinflammation, we employed PLX3397, a CSF1R inhibitor known to reduce microglial numbers and activity [54]. This intervention aimed to assess whether suppressing microglial overactivity could improve depressive‐like behaviors in HSP60 cKO mice. The results showed that PLX3397 treatment significantly ameliorated depressive‐like symptoms, underscoring the critical role of microglial regulation in maintaining synaptic health and emotional stability in the context of HSP60 deficiency.

However, some limits still existed in this study. A significant limitation of this study is its exclusive use of male mice, which may constrain the broader applicability of the results to females. Sex differences in neurobiology, including variations in energy metabolism, stress responses, and synaptic plasticity, are well‐established and play a significant role in shaping the pathophysiology of neurological disorders [55]. Such differences might influence the role of microglial HSP60 and its associated pathways in the context of depression. To gain a more comprehensive insight, future research should include both male and female mice to assess the sex‐specific contributions of microglial HSP60 in synaptic pruning and its broader relevance to depressive disorders. Furthermore, another notable constraint of this study pertains to the lack of regional specificity, as the analysis was conducted at the level of the entire cortex. While the cortex plays a pivotal role in numerous cognitive and emotional processes, it is a heterogeneous structure composed of distinct functional subregions, each potentially contributing in idiosyncratic ways to the pathophysiology of depression. For instance, the medial prefrontal cortex (mPFC), a region heavily implicated in the regulation of mood and stress responses, may exhibit divergent patterns of microglial activation and synaptic pruning relative to other cortical areas. By adopting a global cortical approach, we may have inadvertently obscured these region‐specific nuances in microglial dynamics. Consequently, future investigations should aim to dissect the effects of HSP60 deletion in more localized cortical subregions, such as the mPFC, in order to elucidate how microglial HSP60 modulates synaptic plasticity and neuroinflammation in a spatially confined manner. Such a refined, region‐specific approach would provide profound insights into the role of HSP60 in depressive pathophysiology and could unveil novel, targeted therapeutic strategies for specific brain regions.

## 5 Conclusion

In summary, our study unveils microglial HSP60 as a regulator of mood, with its deficiency leading to depressive‐like behaviors potentially via the microglial phagocytosis of excitatory synapses, causing synaptic dysfunction. Microglial‐specific HSP60 knockout male mice displayed overactive microglia, reduced glutamate, and disrupted synaptic plasticity. Notably, PLX3397 reversed these effects, suggesting the fundamental role of microglial HSP60 in depression.

## Author Contributions

Conceptualization: Weifen Li and Xiuyan Yang. Methodology: Weifen Li and Jinlong Chang. Investigation and Analysis: Weifen Li, Wenhui Zhu, and Liusuyan Tian. Writing: Wenhui Zhu. Supervision: Weifen Li. All authors have read and agreed to the published version of this manuscript.

## Ethics Statement

All experimental procedures were performed according to the protocols approved by the Institutional Animal Care and Use Committee of the Peking University Shenzhen Graduate School, and the animal ethics committee authorization number is AP0013004.

## Conflicts of Interest

The authors declare no conflicts of interest.

## Data Availability

The mass spectrometry proteomics data have been deposited to the ProteomeXchange Consortium (http://proteomecentral.proteomexchange.org) via the iProX partner repository [56, 57] with the dataset identifier PXD059322.
